# Early Mortality during Initial Treatment of Tuberculosis in Patients Co-Infected with HIV at the Yaoundé Central Hospital, Cameroon: An 8-Year Retrospective Cohort Study (2006-2013)

**DOI:** 10.1371/journal.pone.0132394

**Published:** 2015-07-27

**Authors:** Jean Joel R. Bigna, Jean Jacques N. Noubiap, Ako A. Agbor, Claudia S. Plottel, Serge Clotaire Billong, André Patrick R. Ayong, Sinata Koulla-Shiro

**Affiliations:** 1 Department of Epidemiology and Public Health, Pasteur Center of Cameroon, Yaoundé, Cameroon; 2 Department of Medicine, Groote Schuur Hospital and University of Cape Town, Cape Town, South Africa; 3 Medical Diagnosis Center, Yaoundé, Cameroon; 4 Faculty of Medicine and Biomedical Sciences, University of Yaoundé 1, Yaoundé, Cameroon; 5 Department of Medicine, NYU-Langone Medical Center, New York, New York, United States of America; 6 National AIDS control committee, Ministry of Public Health, Yaoundé, Cameroon; 7 Faculty of Medicine and Pharmaceutic Sciences, University of Douala, Douala, Cameroon; 8 Infectious Diseases Unit, Yaoundé Central Hospital, Yaoundé, Cameroon; Chinese Academy of Medical Sciences and Peking Union Medical College, CHINA

## Abstract

**Background:**

Understanding contributors to mortality during the initial phase of tuberculosis (TB) treatment in patients co-infected with HIV would guide targeted interventions to improve survival. The aim of this study was to ascertain the incidence of death during the initial 2 months (new cases) and 3 months (retreatment cases) of TB treatment and to assess correlates of mortality in HIV co-infected patients.

**Methods:**

We conducted a hospital-based retrospective cohort study from January 2006 to December 2013 at Yaoundé Central Hospital, Cameroon. We reviewed medical records to identify co-infected TB/HIV inpatients aged 15 years and older who died during TB treatment. Death was defined as any death occurring during TB treatment, as per World Health Organization recommendations. We collected socio-demographic, clinical and laboratory data. We conducted multivariable logistic binary regression analysis to identify factors associated with death during the intensive phase of TB treatment. Magnitudes of associations were expressed by adjusted odds ratio (a*OR*) with 95% confidence interval. A p value < 0.05 was considered statistically significant.

**Results:**

The 99 patients enrolled had a mean age of 39.5 (standard deviation 10.9) years and 53% were male. Patients were followed for 276.3 person-months of observation (PMO). Forty nine patients were died during intensive phase of TB treatment. Death incidence during the intensive phase of TB treatment was 32.2 per 100 PMO. Having a non-AIDS comorbidity (a*OR* 2.47, 95%CI 1.22-5.02, *p* = 0.012), having extra-pulmonary TB (a*OR* 1.89, 95%CI 1.05-3.43, *p* = 0.035), and one year increase in duration of known HIV infection (aOR 1.23, 95%CI 1.004-1.49) were independently associated with death during the intensive phase of TB treatment.

**Conclusions:**

Mortality incidence during intensive phase of TB treatment was high among TB/HIV co-infected patients during TB treatment; and strongly associated with extra pulmonary TB suggesting advanced stage of immunosuppression and non-AIDS comorbidities. Early HIV diagnosis and care and good management of non-comorbidities can reduce this incidence.

## Introduction

Tuberculosis (TB) remains the most common serious opportunistic infection in people infected with human immunodeficiency virus (HIV) in sub-Saharan Africa and is the leading cause of death [1–5]. In Cameroon, in 2012, the prevalence of HIV infection among adults aged 15 to 49 years was 4.3%, and the death rate from HIV was 159 per 100,000 people [6]. It is estimated that 38% of all HIV-infected patients in Cameroon have had active TB at least once in their lifetime [7]. The mortality rate among those co-infected with TB and HIV was 35 per 100,000 persons [7]. Once active TB is diagnosed, pharmacologic treatment is mandatory and should be prescribed in a timely manner. TB treatment comprises two consecutive phases: an initial intensive phase lasting two months for new cases and three months for retreatment cases, followed by a completion phase using fewer anti-tuberculous medications and lasting four and five additional months, respectively [8–10]. Prior studies have noted that death in TB/HIV co-infected patients tends to occur early in the course of TB treatment [11–14]. In Malawi, factors associated with early death in TB complicating HIV included *Mycobacterium tuberculosis* bacteremia [15]. Also in Malawi, a study focused on premature deaths in TB patients found associations with severe malnutrition, age > 35 years, and the presence of HIV co-infection [16]. A more recent study of TB patients in India [17] determined that increasing age and new (as compared to recurrent) TB disease were risk factors for early mortality. We are not aware however, of any study to date designed to investigate why TB/HIV co-infected patients have a greater mortality during intensive phase of TB treatment in sub-Saharan Africa. In a previous study [18], we reported low TB treatment success rates and high mortality in a cohort of TB/HIV co-infected patients in Yaoundé. In the present study, we examine the incidence and socio-demographic, clinical, and laboratory factors associated with death during intensive phase of TB treatment in our cohort of TB/HIV co-infected patients. The identification of relevant factors would potentially allow health policy makers and clinicians to make recommendations to improve the survival of TB/HIV co-infected patients during anti-TB treatment in resource-limited health care settings.

## Materials and Methods

### Design and Population

Our hospital-based retrospective cohort study took place at the Infectious Diseases Unit (IDU) of the tertiary-care Yaoundé Central Hospital (YCH), in Cameroon. Inpatients aged 15 years and older diagnosed with TB from January 1, 2006 to June 30, 2013 who were co-infected with HIV and who died during their TB treatment were included. We excluded all outpatients as more than 70% of those initiating TB therapy as outpatients had incomplete data. We also excluded patients who died before initiation of TB treatment or on the date TB treatment began, cases with missing information on follow-up and missing documentation of outcomes in the IDU/TB registers, and patients with incomplete file information (at least 30% of variables). We reviewed a total of 681 TB yellow cards [8], handwritten medical records, and the TB registry entries of the IDU-YCH. A TB yellow card is an A4 format paper used in the course of TB treatment. Each yellow card documents a patient’s demographic and administrative information (name, sex, age, address, TB registry number), along with the type of TB at the start of treatment (pulmonary versus extra-pulmonary), HIV status, and a calendar with check boxes for each day anti-TB medications are taken.

### Data collection

For new and retreatment cases, we considered the intensive phase as the first two and the initial three months of anti-tuberculous therapy respectively [8, 10]. Five first-line drugs are used in Cameroon for initiating treatment of new and retreatment cases of TB: Rifampicin (RIF), Isoniazid (INH), Ethambutol (EMB), Pyrazinamide (PZA), and Streptomycin (S) [8]. Irrespective of HIV status, the treatment of newly diagnosed TB in Cameroon begins with an intensive phase of a four-drug regimen of INH+RIF+PZA+EMB lasting two months, followed by a two-drug continuation phase with INH+RIF for four months, for a total of six months of treatment. Retreatment cases require eight months of therapy, starting with an intensive phase of two months of five drugs, INH+RIF+PZA+EMB+S followed by one month of INH+RIF+PZA+EMB, after which the continuation phase consist of five more months of a three-drug regimen of INH+RIF+EMB [8].

A death for the purposes of this study was defined as a death from any cause during treatment for tuberculosis as per the World Health Organization’s recommendations [9]. All of our patients were hospitalized for the intensive phase of their TB treatment, resulting in directly observed treatment (DOT). After the intensive phase patients were permitted to continue their treatment while residing at home. For those patients who died outside a hospital setting, confirmation of death was obtained by phoning the contact person listed in the patient’s medical record. If it was not possible to reach that contact person to confirm the death, the patient was considered as lost to follow-up. As described in detail previously [18], we have collected socio-demographic, clinical, and laboratory data. National recommendations [8] adopted from the WHO Standard International Definitions [9] were used to classify TB status at the time of diagnosis and the type of TB clinical presentation.

### Data analysis

Data was extracted, coded, entered, and analyzed using the Statistical Package for Social Science (SPSS) version 21.0 for Windows (IBM Corp. Released 2012. IBM SPSS Statistics for Windows, Version 21.0. Armonk, NY: IBM Corp.). Continuous variables were expressed as the mean with standard deviation (SD) or median with interquartile range (IQR). Categorical variables were expressed as numbers with percentages (%). Death incidence ratio was analyzed using Windows Program for Epidemiologists version 11.25 and reported with Fisher’s exact 95% confidence interval (95%CI). All follow-ups were censored at three months and two months for retreatment and new cases respectively. This is because the intensive phase of TB treatment is defined as the first three months of therapy for retreatment of TB and the initial two months for new TB cases [10].

Multiple imputation was used to handle missing data, creating a new data set, which was the average of twenty data sets of imputed values imputation [19, 20]. All variables used to search for risk factors of death and outcome variable (death) were included in the imputation model. To limit the loss in power to no more than 1% during imputation, we have done 20 imputations [21]. We have used automatic imputation method without constraints. Continuous and categorical missing data were imputed by linear regression and logistic regression models respectively. Fraction of missing information was lower, between 0.002 and 0.158, then chosen number of imputations were largely adequate [21].

Firstly, we conducted univariate binary logistic regression analysis. After this, we performed multivariable (including all variables tested in univariate analysis) binary logistic regression analysis to identify independent predictors of death during intensive phase of TB treatment. We also performed these univariate and multivariable analyses with categorization of all continuous data (age, duration of know HIV infection, body weight, white blood cell, hemoglobin level, CD4 lymphocytes count). A p value < 0.05 was considered statistically significant. Odds ratios (*OR*) were reported with a 95%CI.

### Ethics statement

We obtained ethical clearance from the Review Board of the Faculty of Medicine and Biomedical Sciences, University of Yaoundé 1, Cameroon. An administrative authorization of the Yaoundé Central Hospital was also obtained before data collection. All data were anonymously collected. Patients’ records and information were anonymized and de-identified prior to analysis.

## Results

**Fig 1:**
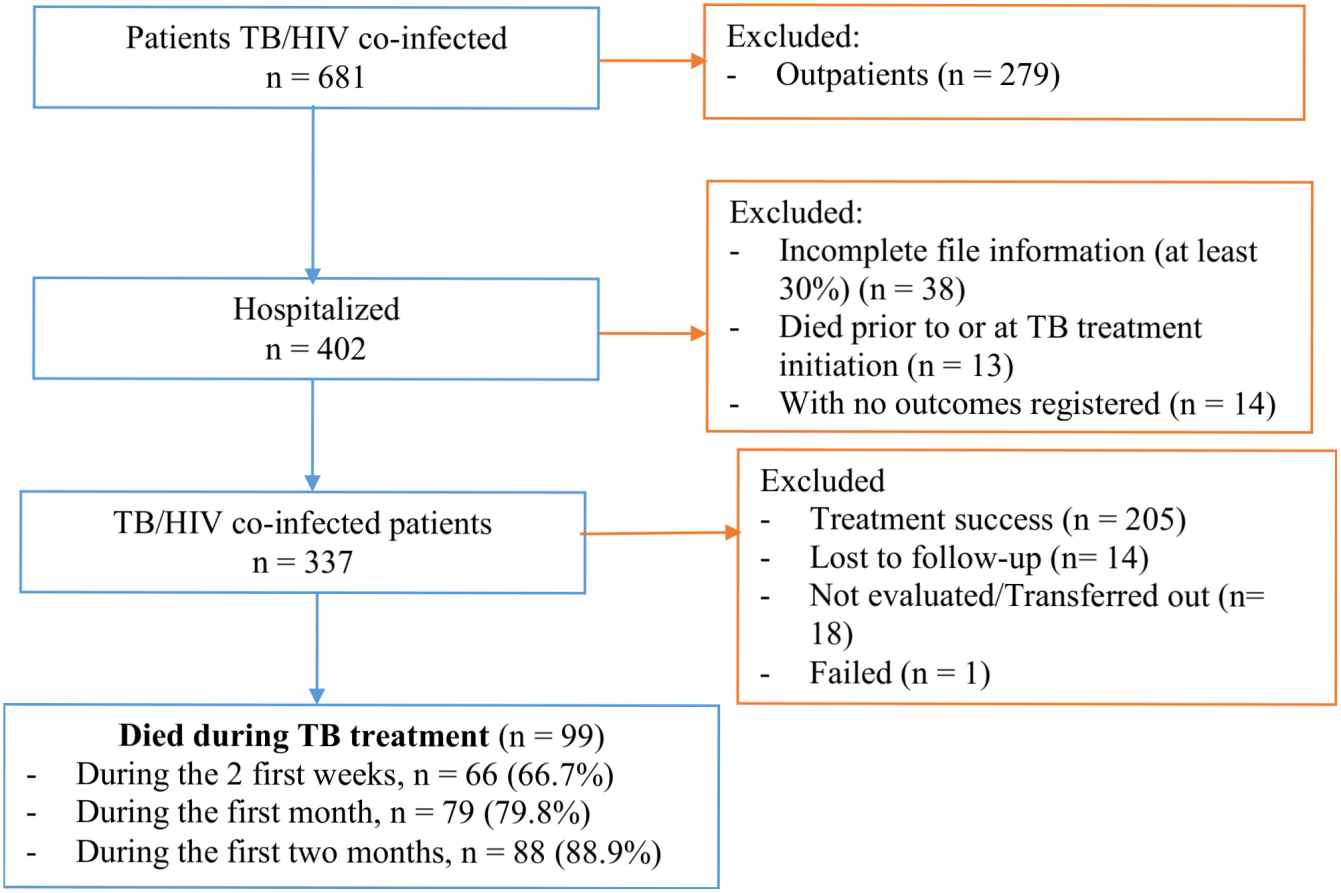
Enrollment of study participants.

Of 337 hospitalized patients, aged 15 years or more with TB who were also HIV positive, we identified 99 patients who died during TB treatment (Fig 1).

### Socio-demographic, clinical and laboratory description of study population

Table 1 presents the socio-demographic, clinical, and laboratory characteristics of our study population (n = 99). The mean age was 39.5 years (SD 10.9). Slightly more than half of the patients were male (n = 52), most had achieved a secondary or tertiary level of higher level of education (n = 69), nearly two-thirds lived alone (n = 60), and the majority resided in an urban area (n = 86) of Cameroon. Most of the TB cases had been diagnosed before 2010 (n = 70), had smear-positive pulmonary TB (n = 41), and represented new cases (n = 88). The mean duration of known HIV infection was 6.1 years (SD 15.6) and the cohort’s mean body weight was 50.0 kg (SD 11.2). Another AIDS-defining infection (besides TB) was present in 27%; one fifth of the cohort had non-AIDS medical comorbidities (n = 20). About one third of our cohort was not taking CPT (n = 32) and not taking ART (n = 36). The median white blood cell count was 4,700 cell/mm^3^ (IQR 3050–8050), the median CD4 lymphocyte count was 42 cell/mm^3^ (IQR 15–101), and the median hemoglobin level was 8 g/dl (IQR 6–9).

**Table 1:** Socio-demographic, clinical, and immunological description of study population.

Variables	Death during intensive phase (n = 89)	Death after intensive phase (n = 10)	All (N = 99)
**SOCIO-DEMOGRAPHIC PROFILE**			
Sex			
- Male	47 (53)	5 (50)	52 (53)
- Female	42 (47)	5 (50)	47 (47)
Age, years			
- Mean	40.1 (10.8)	34.3 (11.0)	39.5 (10.9)
- Median	38 (33–48)	36 (26–39)	37 (32–48)
Level of education			
- Primary/No formal	29 (33)	1 (10)	30 (30)
- Secondary/Tertiary	60 (67)	9 (90)	69 (70)
Marital status			
- Alone (single/widowed/ divorced)	53 (60)	7 (70)	60 (61)
- Married/Cohabitating	36 (40)	3 (30)	39 (39)
Residence			
- Rural	12 (13)	1 (10)	13 (13)
- Urban	77 (87)	9 (90)	86 (87)
**CLINICAL PROFILE**			
Year of TB diagnosis			
- 2006–2009	64 (72)	6 (60)	70 (71)
- 2010–2013	25 (28)	4 (40)	29 (29)
TB clinical presentation*			
- Mixed (Pulmonary + EP TB)	6 (7)	2 (20)	8 (8)
- EP TB only	26 (29)	5 (50)	31 (31)
- SNP TB only	17 (19)	2 (20)	19 (19)
- SPP TB only	40 (45)	1 (10)	41 (41)
Status at TB diagnosis			
- Retreatment case	11 (12)	0	11 (11)
- New case	78 (88)	10 (100)	88 (89)
Body weight^α^ (Kg)			
- Mean	50.0 (11.3)	49.7 (10.7)	50.0 (11.2)
- Median	48 (42–55)	49 (43–59)	48 (42–55)
Duration of known HIV infection, months			
- Mean	5.4 (13.9)	12.4 (26.8)	6.1 (15.6)
- Median	0.5 (0.1–3.2)	0.5 (0.1–11.8)	0.5 (0.1–3.6)
Presence of another AIDS-related disease			
- Yes	23 (26)	4 (40)	27 (27)
- No	66 (74)	6 (60)	72 (73)
Presence of another non-AIDS comorbidity			
- Yes	16 (18)	4 (40)	20 (20)
- No	73 (82)	6 (60)	79 (80)
Cotrimoxazole prophylactic therapy			
- No	30 (34)	2 (20)	32 (32)
- Yes	59 (66)	8 (80)	67 (68)
Antiretroviral therapy			
- No	33 (37)	3 (30)	36 (36)
- Yes	56 (63)	7 (70)	63 (64)
**LABORATORY PROFLE**			
White blood cell count^β^, cells/mm^3^			
- Mean	6,895 (7,836)	6,168 (4,843)	6,815 (7,551)
- Median	4,700 (3,100–8,000)	4,450 (2,500–10,000)	4,700 (3,050–8,050)
Hemoglobin level^β^, g/dl			
- Mean	7.8 (2.5)	8.1 (2.0)	7.8 (2.5)
- Median	8 (6–9)	8 (7–9)	8 (6–9)
CD4 Lymphocytes count^β^, cells/mm^3^			
- Mean	71.5 (84.3)	66.0 (71.3)	70.9 (82.7)
- Median	42 (15–97)	29 (14–143)	42 (15–101)

Data are n (%), mean (standard deviation) and median (interquartile range).

* SPP: smear positive pulmonary, SNP: smear negative pulmonary, EP: extra pulmonary.

TB: tuberculosis

^α^ = 9 missing data,

^β^ = 7 missing data

### Incidence of death during intensive phase of TB treatment

**Fig 2:**
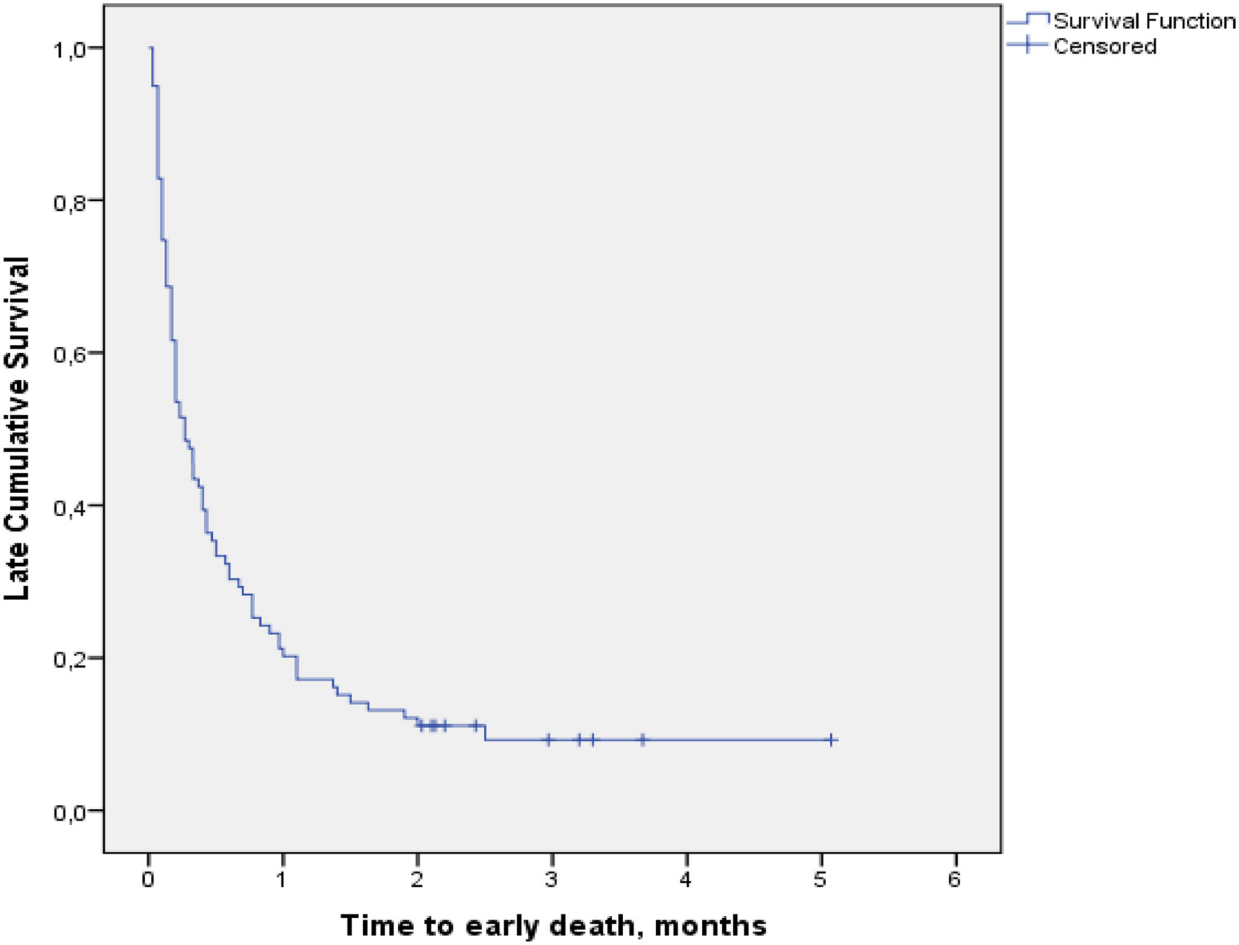
Kaplan-Meier survival curve of cumulative survival over time during tuberculosis treatment of patients co-infected with HIV.

Patients were followed for 276.3 person-months of observation with median duration of 8.1 days (IQR 3.0–24.9) and mean of 20.4 days (SD 27.9). Among the 99 patients who died during TB treatment, 89 died during the intensive phase of TB treatment. Death incidence during the intensive phase of TB treatment was 32.2 per 100 persons-months of observation. Fig 2 presents a Kaplan-Meier survival curve over the course of TB treatment. The slope of the curve is steepest for the first month and flattens out after the third month, indicating a high rate of death during the first two months, most notably during the first (Fig 2).

### Factors associated with death during intensive phase of TB treatment

In our univariate analysis, two factors associated with death during the intensive phase of TB treatment were extra-pulmonary TB at presentation (*OR* 1.84, 95%CI 1.11–3.05, *p* = 0.018) and the presence of any non-AIDS comorbidity (*OR* 1.85, 95%CI 1.07–3.20, *p* = 0.027). Independent factors associated with death during intensive phase of TB treatment were having non-AIDS comorbidity (a*OR* 2.47, 95%CI 1.22–5.02, *p* = 0.012), having extra-pulmonary TB ((a*OR* 1.89, 95%CI 1.05–3.43, *p* = 0.035) compared to localized pulmonary TB, and one year increase in duration of known HIV infection (aOR 1.23, 95%CI 1.004–1.49). These results are reported in Table 2.

**Table 2:** Factors associated with death during the intensive phase of tuberculosis treatment.

Variables		Univariate analysis		Multivariable analysis	
		p	Odds ratios (95%CI)	p	Adjusted odds ratios (95%CI)
**Sex**	Male	.786	0.94 (0.62–1.43)	.707	0.89 (0.49–1.62)
	Female		1		
**Age, years**	Continuous	.241	1.01 (0.99–1.03)	.049	1.03 (1.00–1.05
**Level of education**	Primary/No formal	.763	1.07 (0.69–1.67)	.814	0.94 (0.56–1.57)
	Secondary/Tertiary		1		
**Marital status**	Alone (single/widowed/ divorced)	.913	0.97 (0.64–1.50)	.406	1.30 (0.70–2.39)
	Married/Cohabitating		1		
**Area of residence**	Rural	.111	1.65 (0.89–3.04)	.109	1.89 (0.87–4.09)
	Urban		1		
**Year of TB diagnosis**	2006–2009	.657	1.11 (0.70–1.77)	.646	1.15 (0.63–2.10)
	2010–2013		1		
**Clinical presentation** *	SPP TB only		1		
	SNP TB only	.749	1.11 (0.60–2.04)	.265	1.53 (0.73–3.21)
	EP TB only	.018	1.84 (1.11–3.05)	.035	1.89 (1.05–3.43)
	Mixed (Pulmonary + EP TB)	.644	0.81 (0.33–1.97)	.971	1.02 (0.36–2.84)
**Status at TB diagnosis**	New	.942	0.98 (0.52–1.84)		
	Retreatment		1	.449	0.74 (0.34–1.60)
**Duration of know HIV infection, years**	Continuous^α^	.300	0.91 (0.77–1.08)	.045	0.81 (0.67–0.996)
**Body weight, Kg**	Continuous^α^	.631	1.01 (0.99–1.02)	.795	0.997 (0.98–1.02)
**Presence of another AIDS opportunistic infection**	Yes	.196	1.37 (0.85–2.21)	.337	1.33 (0.74–2.37)
	No		1		
**Presence of non-AIDS comorbidity**	Yes	.027	1.85 (1.07–3.20)	.012	2.47 (1.22–5.02)
	No		1		
**Cotrimoxazole prophylactic therapy**	Yes		1		
	No	.363	0.81 (0.52–1.27)	.629	0.88 (0.52–1.50)
**Antiretroviral therapy**	Yes		1		
	No	.899	1.03 (0.67–1.58)	.148	1.48 (0.87–2.53)
**White blood cell count, cells/mm** ^**3**^	Continuous	.558	1.00 (1.00–1.00)	.448	1.00 (1.00–1.00)
**Hemoglobin level, g/dl**	Continuous^α^	.230	0.95 (0.87–1.04)	.513	0.96 (0.86–1.08)
**CD4 lymphocytes count, cells/mm** ^**3**^	Continuous	.442	1.00 (0.998–1.004)	.906	1.00 (0.997–1.003)

^α^: These three continuous data had negative coefficient of correlation

* SPP: smear positive pulmonary, SNP: smear negative pulmonary, EP: extra-pulmonary

TB: tuberculosis

After categorization of all continuous data, in multivariable analysis, having extra-pulmonary TB (adjusted *OR* 2.19, 95%CI 1.51–4.17, *p* = 0.017) as compared to localized pulmonary TB and having non-AIDS comorbidity (adjusted *OR* 2.23, 95%CI 1.12–4.46, *p* = 0.023), was associated with death during intensive of TB treatment. Duration of known HIV infected was not associated with early death when is categorized. These results were reported in Table 3.

**Table 3:** Factors associated with death during the intensive phase of tuberculosis treatment with all variables categorized.

Variables		Univariate analysis		Multivariable analysis	
		p	Odd ratios (95%CI)	p	Adjusted odd ratios (95%CI)
**Sex**	Male	.786	0.94 (0.62–1.43)	.196	0.67 (0.36–1.23)
	Female		1		
**Age, years**	20–34	.241	1.01 (0.99–1.03)	.600	0.83 (0.40–1.69)
	35–49	.763	1.07 (0.69–1.67)	.758	0.90 (0.45–1.80)
	50 and more		1		
**Level of education**	Primary/No formal	.763	1.07 (0.69–1.67)	.997	1.00 (0.59–1.70)
	Secondary/Tertiary		1		
**Marital status**	Alone (single/widowed/ divorced)	.913	0.97 (0.64–1.50)	.784	1.09 (0.58–2.06)
	Married/Cohabitating		1		
**Area of residence**	Rural	.111	1.65 (0.89–3.04)	.321	1.50 (0.67–3.36)
	Urban		1		
**Year of TB diagnosis**	2006–2009	.657	1.11 (0.70–1.77)	.791	1.09 (0.57–2.11)
	2010–2013		1		
**Clinical presentation** *	SPP TB only		1		
	SNP TB only	.749	1.11 (0.60–2.04)	.095	1.97 (0.89–4.36)
	EP TB only	.018	1.84 (1.11–3.05)	.017	2.19 (1.51–4.17)
	Mixed (Pulmonary + EP TB)	.644	0.81 (0.33–1.97)	.875	1.09 (0.39–3.06)
**Status at TB diagnosis**	New	.942	0.98 (0.52–1.84)		
	Retreatment		1	.336	1.47 (0.67–3.196)
**Duration of know HIV infection, years**	< 1		1		
	≥ 1	.394	0.76 (0.40–1.43)	.186	0.56 (0.24–1.31)
**Body weight, Kg**	< 50	.939	1.02 (0.67–1.56)	.727	1.10 (0.65–1.84)
	≥ 50		1		
**Presence of another AIDS opportunistic infection**	Yes	.196	0.73 (0.45–1.18)	.304	1.37 (0.75–2.47)
	No		1		
**Presence of non-AIDS comorbidity**	Yes	.027	1.85 (1.07–3.20)		
	No		1	.023	2.23 (1.12–4.46)
**Cotrimoxazole prophylactic therapy**	Yes		1		
	No	.363	0.81 (0.52–1.27)	.769	1.09 (0.62–1.92)
**Antiretroviral therapy**	Yes		1		
	No	.899	1.03 (0.67–1.58)	.125	0.65 (0.38–1.13)
**White blood cell count, cells/mm** ^**3**^	< 4.000	.530	0.86 (0.54–1.37)	.712	0.90 (0.52–1.56)
	> 10.000	.672	1.14 (0.62–2.11)	.982	0.99 (0.47–2.09)
	4.000–10.000		1		
**Hemoglobin level, g/dl**	< 8	.800	1.06 (0.69–1.62)	.565	0.86 (0.51–1.45)
	≥ 8		1		
**CD4 lymphocytes count, cells/mm** ^**3**^	< 50	.434	0.72 (0.31–1.64)	.552	0.74 (0.27–2.03)
	50–199	.477	0.73 (0.31–1.74)	.346	0.60 (0.21–1.74)
	≥ 200		1		

* SPP: smear positive pulmonary, SNP: smear negative pulmonary, EP: extra-pulmonary

TB: tuberculosis

## Discussion

Our study aimed to determine the incidence of death and factors associated with mortality during the intensive phase of TB treatment in TB/HIV co-infected inpatients. There was a high mortality rate during the intensive phase of TB treatment among TB/HIV co- infected patients. The presence of extra-pulmonary TB at treatment initiation, non-AIDS medical comorbidities, and increasing duration of known HIV infection were independent risk factors for death.

Our study found a high mortality rate (90%, n = 89) of death during intensive phase of TB treatment among patients co-infected with TB and HIV. This finding is consistent with other studies reporting high rates of early death in TB/HIV co-infected people during ongoing TB treatment [12–14]. As we demonstrated in a recently published study of the IDU-YCH cohort [18] and in other studies in Africa [14, 22, 23], most patients present at an advanced stage of immunosuppression. Prescribing ART close to the time of TB treatment initiation in a person at an advanced stage of immunosuppression can lead to immune reconstitution inflammatory syndrome (IRIS) [24–27]. It is well established that mortality increases in the setting of IRIS [25–27]. That fact is a possible explanation for some of the deaths during the intensive initiation phase (early death) of TB treatment in patients infected with HIV. Controversy remains as to the optimal timing of ART initiation in TB/HIV co-infection. Some studies recommend early initiation of ART [28, 29]; a more recent prospective randomized trial however, advocates for delayed ART initiation [30]. As the cause of death was not ascertained in our study we cannot determine how many patients did in fact have IRIS or IRIS-related mortality.

Our study also reveals that a diagnosis of extra-pulmonary TB at presentation is a risk factor for death during the intensive phase. Due to the difficultly in diagnosing extra-pulmonary TB in our resource-limited clinical setting, often compounded by patients’ limited financial resources that would allow for timely targeted diagnostic testing, such a diagnosis tended to be made late, at a more advanced stage of disease. Also, extra-pulmonary TB is a marker of more advanced host immunosuppression, as compared to pulmonary TB that remains confined to the respiratory system. The same pathophysiologic process related to increasing immunosuppression is invoked as IRIS, which favors death [25–27], occurs mainly at the advanced stage of immunosuppression [24–27].

The presence of a non-AIDS medical comorbidity was associated with death during the intensive phase of TB treatment in our study. This is a novel finding. We have not found any published study implicating this clinical factor in mortality during intensive of TB treatment. One likely explanation is that comorbidities further compromise the health of TB/HIV co-infected patients, or their response to the anti-tuberculous medication, or both, thus contributing to premature death. The care of HIV-infected patients with tuberculosis and non-AIDS related comorbidities should therefore be carefully initiated as soon as possible, with close attention to identification and treatment of non-AIDS illnesses. The plan of care especially during the intensive phase of anti-tuberculous medication prescription, should also focus on and address the comorbidity. Identification of patients with this risk factor, and its optimal management could ameliorate the overall survival of TB/HIV co-infected patients undergoing TB treatment. Another likely contributor to the increased mortality in patients with a non-AIDS comorbidity is that the polypharmacy of multiple drugs (non-AIDS comorbidity drugs, plus TB drugs and/or ART and CPT), along with the required multidrug antituberculous regimen could result in less adherence to treatment [31–34], to increased drug-related adverse events, and to decreases in bioavailability of some drugs [35, 36].

We also found as a factor associated with early death, increasing duration to be known as HIV-infected. This result was found in another study [37]; and several possible reasons can explain. Life expectancy may decrease gradually as the duration of infection increases. Patients who have a long duration of HIV infection develop more resistance to antiretroviral treatment.

Our study has some limitations to consider. It captured patients undergoing treatment for tuberculosis over a consecutive 90-month period, from January 1, 2006 through June 30, 2013. The Cameroonian Ministry of Public Health issued new guidelines for prescribing ART in 2012. Prior to 2012, it was recommended that ART therapy in the setting of HIV be started when the CD4 cell count dropped to less than 200 cells/mm^3^; the 2012 revision instead advised prescribing ART once the CD4 cell count reached 350 cells/mm^3^. This likely lead to more of our patients being started on ART later in the study period; tuberculosis guidelines however, remained unchanged for the entire period of our study. Some data for body weight, hemoglobin level, white blood cell count, and CD4 count were missing. However, multiple imputations have been used to handle the missing data. Whatever the underlying cause, the missing data underscores the challenges of conducting “real-life” clinical research in resource-limited settings, like Cameroon and other sub-Saharan countries. Our results may, therefore, not be generalizable to all patients with TB/HIV, and particularly to those receiving their treatment on an ambulatory basis. After starting treatment in hospital, patients continued their TB as outpatients; it is thus possible that we underestimated the actual overall death rate as some patients were lost to follow-up. This study thus provides at least a conservative assessment of mortality while providing knowledge of risks and preliminary information to guide future research.

## Conclusions

In our setting, among patients co-infected with TB and HIV undergoing TB treatment, the incidence of death during intensive phase of TB treatment was high. The independent factors associated with mortality during intensive of TB treatment were easily identifiable clinical ones, having extra-pulmonary TB and having non-AIDS comorbidity. Timely consideration and identification of extra-pulmonary TB can help to reduce early death by allowing for prompt initiation of TB treatment at a relatively earlier stage of immunosuppression. Treatment of non-AIDS comorbidities and optimal control of chronic ones, along with reduction in the amount of medications prescribed in order to include only essential pills are straightforward measures that may help to reduce early death. Further studies focused on mortality during initial TB treatment are needed and should include investigation into the causes of death in order to better determine if early deaths are due to inadequate TB control, or to other processes including, but not limited to drug toxicities, IRIS, or worsening of comorbid illnesses.

## Supporting Information

S1 Data Data of the 99 patients included in the study. (XLSX)
